# Does depression affect the association between prosocial behavior and anxiety? A cross-sectional study of students in China

**DOI:** 10.3389/fpubh.2023.1274253

**Published:** 2023-10-10

**Authors:** Xiyan Zhang, Tao Lv, Gerard Leavey, Na Zhu, Xin Li, Yan Li, Yanhua Chen

**Affiliations:** ^1^Clinical Research Center for Mental Disorders, Shanghai Pudong New Area Mental Health Center, School of Medicine, Tongji University, Shanghai, China; ^2^People’s Hospital of Deyang, Deyang, China; ^3^Bamford Centre for Mental Health and Wellbeing, Ulster University, Coleraine, United Kingdom; ^4^Shanghai Mental Health Center, Shanghai Jiao Tong University School of Medicine, Shanghai, China

**Keywords:** prosocial behavior, anxiety, depression, adolescent, emotion

## Abstract

**Background:**

A growing number of studies have suggested that adolescents’ prosocial behavior can protect against depression and anxiety. It is known that anxiety and depression are often comorbid. However, it remains unclear if when depression is present, prosocial behavior remains protective against anxiety, and if when anxiety is present, prosocial behavior remains protective against depression. The purpose of this study was to determine the association of anxiety and depressive with prosocial behavior.

**Methods:**

A large representative sample of middle-school students was recruited for a cross-sectional study and completed standardized instruments (the Children’s Depression Inventory (CDI), Screen for Child Anxiety Related Emotional Disorders–Child version (SCARED-C), and Strengths and Difficulties Questionnaire (SDQ)). We used structural equation modeling (SEM) to examine the protective effect of prosocial behavior against anxiety when depression was present.

**Results:**

A survey of 3,510 students was conducted, and the final analysis included 3,169 students, comprising 1,616 boys (51.0%) and 1,553 girls (49.0%), with a mean age of 13.09 years (SD = 1.31, range 11–16).The prevalence rates of anxiety and depression in early adolescents were 31.6 and 16.7%, respectively. More than two-thirds of depressed adolescents had comorbid anxiety, while more than one-third of anxious adolescents had comorbid depression. Regression models showed that compared with depressed adolescents, adolescents without depressive symptoms exhibited a significant negative correlation between prosocial behaviors and anxiety and depression (*β* = −0.01, *p >* 0.01, *β* = −0.06, *p >* 0.01; *β* = −0.11, *p <* 0.01, and *β* = −0.17, *p <* 0.01). There was no difference in the relationship between prosocial behavior and depression between anxious and non-anxious adolescents (*p >* 0.05).

**Conclusion:**

Anxiety and depression are common in adolescence and are often comorbid disorders. However, the comorbidity is not symmetrical. Specifically, the protective effect of prosocial behavior against anxiety is weaker in depressed adolescents. Findings are discussed in light of related research and theory, and insights for intervention programs and future research are presented.

## 1. Introduction

The prevalence rates of mental health problems have increased among adolescents worldwide, as high as 20.0% (1, 2). If left untreated, they tend to persist into adulthood and are associated with a range of adverse outcomes (3). Mental health problems account for 16% of the disease and injury burden among adolescents aged 10–19 years worldwide, according to statistics released by WHO in 2019. Thus, promoting mental health among adolescents is a public health priority (4).

Anxiety and depression are the most prevalent mental health problems in adolescence. Nearly 40% of adolescents reported having experienced anxiety symptoms, and 8.3% qualified for severe anxiety-related impairment (5). Anxiety is the sixth leading cause of disease and disability for adolescents aged 11–14 years and the ninth leading cause for those aged 15–19 years (6). A meta-analysis indicated that the prevalence of depressive symptoms in adolescents was18.4% before 2000 and was 26.3% after 2016 in China (7). More than half of adolescents who commit suicide may have had a depressive disorder at the time of death (8), and depression in adolescents is a primary risk factor for suicide (8). The high prevalence of anxiety and depression in adolescents and the high rates of disability require a focus on adolescent anxiety and depression.

Anxiety and depression often co-occur (9, 10). Diagnostic comorbidity rates reported in some clinical samples were as high as 75% (11, 12). Approximately 25–50% of depressed youth have anxiety, and approximately 10–15% of anxious youth have comorbid depression (13). Depressed youths with comorbid anxiety tend to have more severe depressive symptoms than no anxious depressed youths (13). Thus, the symptoms may be magnified in children and adolescents with comorbidities (13). However, individuals with a comorbid disorder also exhibit some unique characteristics. One study found that depressed youths tended to report high levels of depression and anxiety, while anxious youths tended to report relatively low levels of depression (14). Comorbidity may result in greater overall impairment, higher rates of suicide attempts and worse treatment outcomes (15, 16). A better understanding of the relationship between anxiety and depression is crucial for preventing comorbidities and mitigating their impact in depressed/anxious adolescents. Anxiety and depression share various risk and protective factors (17), individually, they might not exert the same level of influence on outcomes. Exploring risk and protective factors and determining their effects are important to prevent anxiety and depression in adolescents.

Prosocial behavior, often referred to as sociability, is a complex amalgamation of different types of social interactions (18). Prosocial behaviors are intended to benefit others, including helping, caring, cooperating, sharing, sympathizing and comforting (19). Recent meta-analyzes indicated that prosocial activities have a positive effect on well-being and reduce negative emotions. Prosocial behavior is socially respected, and the social rewards it accrues may, consequently, improve mood (20). Conversely, prosocial behavior may protect against negative feelings (21), depression and anxiety (22, 23). These studies have not considered the high rates of comorbidity observed. However, epidemiological studies suggest that comorbidity is universal, not exceptional; therefore, solely exploring the association of prosocial behaviors with a single mental health problem may not be comprehensive. It is essential to determine the association of depression and anxiety with prosocial behaviors in adolescents to elucidate internalizing problems in adolescents as well as to implement more optimal interventions for anxiety and depression. The present study aimed to investigate (a) the association between prosocial behavior and anxiety when depression was present, and (b) the association between prosocial behavior and depression when anxiety was present.

## 2. Methods

### 2.1. Subjects and procedure

This cross-sectional study used a stratified random cluster sampling method to recruit students from junior high schools in the Pudong New Area, Shanghai, China, from September to November 2021. A total of 3,510 participants aged 11–16 years took part in the survey. After excluding the data of participants with invalid questionnaires, 3,169 participants were included in the final analysis, for a response rate of 90.3%.

### 2.2. Ethical approval

Approval for the study was obtained from the Ethics Committee of Shanghai Pudong New Area Mental Health Center. The parents of all participants provided informed consent to participate in the study (PDJWLL2021030).

### 2.3. Measures

#### 2.3.1. Sociodemographic variables

A custom-designed demographic questionnaire was used to collect the participants’ general information, including age, gender, monthly family income, marital relationship of parents (harmony/disharmony), family structure (nuclear family/extended family/other family structures), parenting style (consistent/inconsistent) and relationship with friends (good/acceptable/bad). Family economic status was categorized into three classes according to monthly family income: lower (less than ¥5,000), middle (¥5,000–10,000), and upper (more than ¥10,000).

#### 2.3.2. Depressive symptoms

Depressive symptoms were assessed using the Children’s Depression Inventory (CDI), which consists of 27 items. The CDI is a comprehensive multilayer assessment of depressive symptoms in children and adolescents and has five subscales: negative mood, interpersonal problems, ineffectiveness, anhedonia and negative self-esteem (24, 25). Participants were asked to indicate the presence of 27 problems in the last 2 weeks on a 3-point scale ranging from 0 (none) to 2 (distinct symptom). Higher scores indicate more depressive symptoms. In this study, participants with a cutoff score of 19 or higher were considered to have significant depressive symptoms. The CDI has good internal consistency, with Cronbach’s alpha coefficients ranging from 0.71 to 0.89 (26). The reliability and validity of this test in the Chinese population have been demonstrated (27). The Cronbach’s alpha coefficient of the CDI was good (0.88) in the present study.

#### 2.3.3. Anxiety symptoms

Anxiety symptoms were assessed using the Screen for Child Anxiety Related Emotional Disorders–Child version (SCARED-C), which measures 41 symptoms. Participants are asked how often they had been bothered by each symptom over the previous 3 months. The response options are “almost never,” “sometimes,” and “often,” scored as 0, 1 and 2, respectively. The SCARED-C includes five subscales: generalized anxiety symptoms, separation anxiety symptoms, social anxiety symptoms, panic or somatic symptoms, and school avoidance (28, 29). A total score of 25 or more has been recommended to indicate significant clinical anxiety (30, 31). Good retesting reliability and validity for the SCARED-C have been confirmed in the Chinese population (32). The Cronbach’s alpha coefficient of this questionnaire was 0.84 in the present study.

#### 2.3.4. Prosocial behavior

Prosocial behavior was assessed using the prosocial scale of the Strengths and Difficulties Questionnaire (SDQ), which is a 25-item checklist (33). The SDQ is divided into five scales of five items each: conduct problems, emotional problems, hyperactivity, peer problems, and prosocial behavior (34). The prosocial scale assesses adolescents’ prosocial behavior. Items are rated on a 3-point Likert scale ranging from 0 to 2 (0 = never, 1 = sometimes, 2 = always). Higher scores indicate more prosocial behavior (35). There are three versions of this questionnaire: the parent version, the teacher version and the self-report version (36), and the self-report version was used in this study. The Chinese version of the SDQ has been demonstrated to have good reliability and validity in Chinese adolescents (37). The internal consistency of the prosocial behavior score (Cronbach’s alpha = 0.70) in the present study was sufficient.

### 2.4. Statistical analysis

Descriptive and inferential statistics were calculated using SPSS version 25.0 for Windows (SPSS, Inc., Chicago, IL, United States). AMOS24 (IBM Corporation, Armonk, NY, United States) was used for correlation analysis and regression analysis. Categorical variables are described as frequencies and percentages and were compared using the chi-square test. The internal consistency of the CDI, SCARED-C and the prosocial scale was evaluated using Cronbach’s alpha values. Pearson correlation analyzes were used to analyze relationships between study variables. A *p* value <0.05 was considered statistically significant, and all statistical tests were two-sided.

Structural equation modeling (SEM) was used to examine the relationships of latent variables. Model fit was evaluated using the chi-square test (*χ*^2^/df), root mean square error of approximation (RSMEA), comparative fit index (CFI), incremental fit index (IFI) and Tucker–Lewis index (TLI). Notably, *χ*^2^/df > 2 implies good model fit; RSMEA values <0.05 represent close model fit, and values >0.10 reflect poor model fit (38); and CFI, IFI, and TLI values >0.90 indicate good fit (39). The fit indices should be interpreted collectively. Standardized coefficients (β) were examined with regression path analyzes.

## 3. Results

### 3.1. Demographic characteristics of subjects

A survey of 3,510 students was conducted, and a total of 3,169 (90.3%) students were included in the final analysis. The sample consisted of 1,616 (51.0%) boys and 1,553 (49.0%) girls, with a mean age of 13.09 years (SD = 1.31, range 11–16). Descriptive statistics for all categorical variables are shown in Table 1. Most participants were from nuclear families (61.0%), had middle or upper family economic status (86.2%), maintained good relationships with their friends (62.1%), reported that their parents had a “good” relationship (88.5%), and reported experiencing a consistent parenting style (70.9%).

**Table 1:** Distribution of variables, and association with anxiety and depression among middle school students.

Variables	Total *N* = 3,169		Depression			Anxiety		
	*n*	(%)	*n*	(%)	Value of *p*	*n*	(%)	Value of *p*
Sex								
Male	1,616	(51.0)	268	(16.6)	0.829	436	(27.0)	0.000
Female	1,553	(49.0)	262	(16.9)		564	(36.3)	
Marital relationship of parents								
Harmony	2,803	(88.5)	478	(17.1)	0.181	876	(31.3)	0.310
Disharmony	366	(11.5)	52	(14.2)		124	(33.9)	
Family structure								
Nuclear family	1933	(61.0)	349	(18.1)	0.020	643	(33.3)	0.020
Extended family	955	(30.1)	133	(13.9)		283	(29.6)	
Other family structures	281	(8.9)	48	(17.1)		74	(26.3)	
Family economic situation								
Upper	1,126	(35.5)	200	(17.8)	0.444	366	(32.5)	0.335
Middle	1,606	(50.7)	263	(16.4)		488	(30.4)	
Lower	437	(13.8)	67	(15.3)		146	(33.4)	
Parenting style								
Consistent	2,247	(70.9)	377	(16.8)	0.917	698	(31.1)	0.355
Inconsistent	922	(29.1)	153	(16.6)		302	(32.8)	
Relationship with friends								
Good	1967	(62.1)	336	(17.1)	0.757	605	(30.8)	0.406
Okay	649	(20.5)	103	(15.9)		209	(32.2)	
Bad	553	(17.4)	91	(16.5)		186	(33.6)	
Depression	530	(16.7)	530	(100.0)		375	(70.8)	
Anxiety	1,000	(31.6)	375	(37.5)		1,000	(100.0)	
Both anxiety and depression	375	(11.8)	375	(100.0)		375	(100.0)	

### 3.2. Prevalence and comorbidity rates of anxiety and depression among middle school students

The prevalence rates of anxiety and depression among middle school students were 31.6 and 16.7%, respectively. Boys had a lower prevalence of anxiety than girls (*p < 0.05*) but a similar prevalence of depression (*p > 0.05*). The prevalence rates of anxiety and depression were higher in participants from nuclear families than in those from large families and other types of families, and there were significant differences among family types (*χ*^2^ = 7.80, *p* = 0.020; *χ*^2^ = 7.85, *p* = 0.020). We found no significant difference in other variables (*p > 0.05*). More than two-thirds of students who met the threshold for depression as a primary condition also had anxiety symptoms, while more than one-third of students who met the threshold for anxiety as a primary condition also had depressive symptoms. The comorbidity rate of anxiety and depression was 11.8% among adolescents. See Table 1 for more details.

### 3.3. Correlations between study variables

Table 2 summarized the correlations found between study variables with means and standard deviations for all continuous study variables. Prosocial behavior was negatively associated with anxiety, depression and SDQ-Externalized Problems (*r* = −0.177, *p* < 0.001; *r* = −0.359, *p* < 0.001; *r* = −0.392, *p* < 0.001). SDQ-Externalized Problems was positively associated with anxiety and depression (*r* = −0.452, *p* < 0.001; *r* = −0.615, *p* < 0.001). Anxiety was positively associated with depression (*r* = −0.589, *p* < 0.001).

**Table 2:** Bivariate correlations between and descriptive statistics for all continuous study variables (*r* value).

Variables	1	2	3	4
1.PB	–			
2.Anxiety	−0.177**	–		
3.Depression	−0.359**	0.589**	–	
4.SDQ-Externalized Problems	−0.392**	0.452**	0.615**	–
Mean	7.90	18.26	11.10	4.73
(SD)	(2.11)	(13.65)	(7.66)	(3.31)

PB, Prosocial behavior, **p < 0.001.

### 3.4. Effects of prosocial behavior on anxiety in individuals with depression and on depression in individuals with anxiety

**Figure 1:**
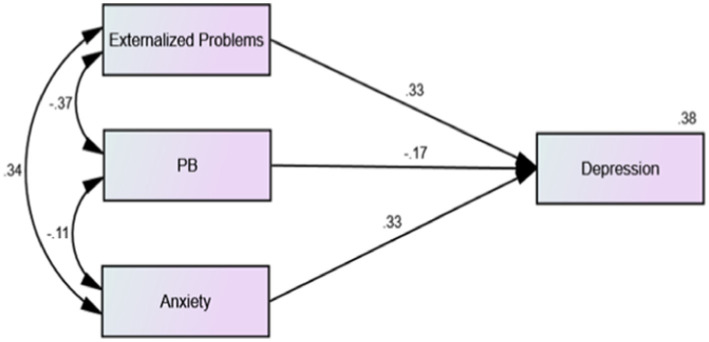
Regression model of external problems, prosocial behavior, anxiety and depression factors of middle school students without depressive symptoms.

**Figure 2:**
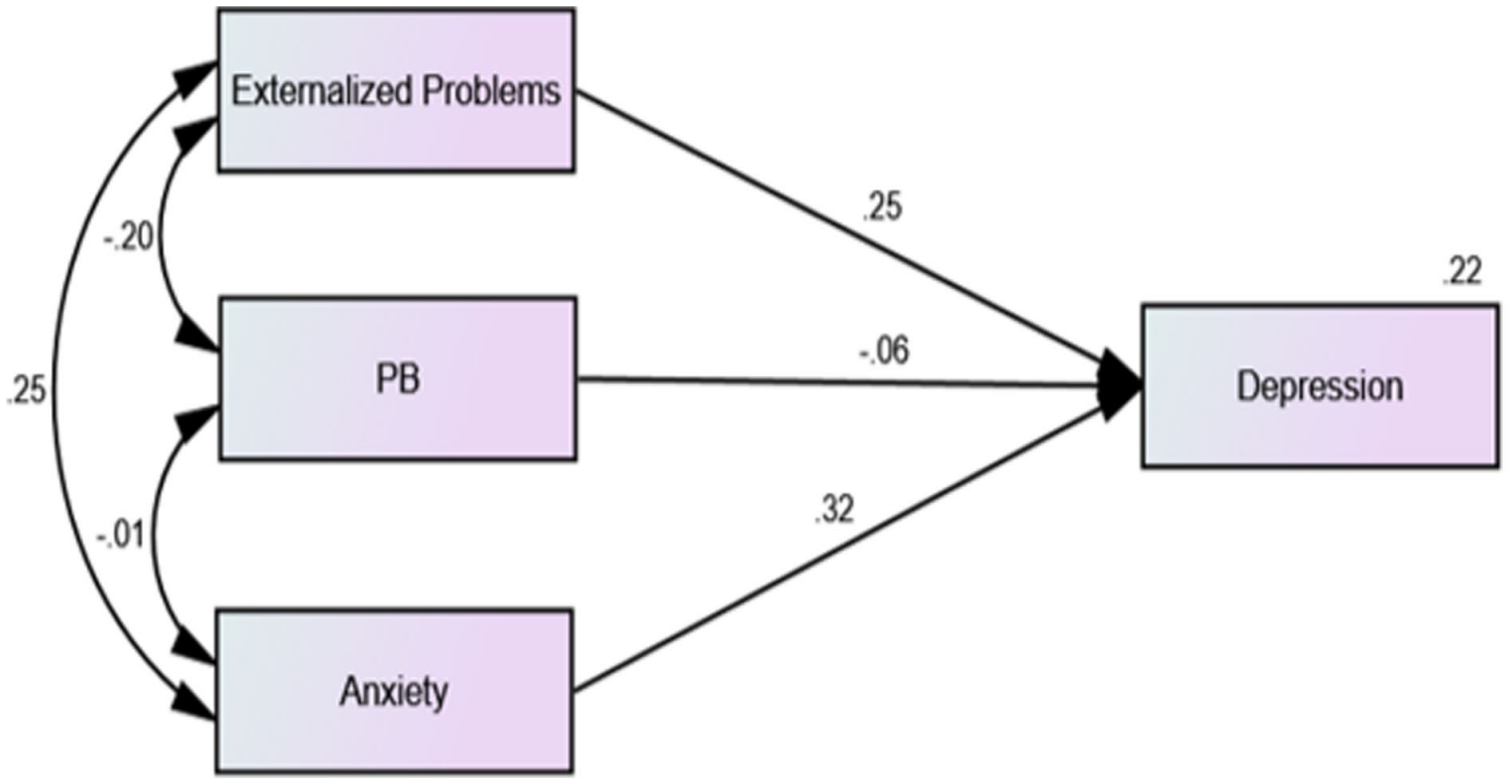
Regression model of external problems, prosocial behavior, anxiety and depression factors of middle school students with depressive symptoms.

**Figure 3:**
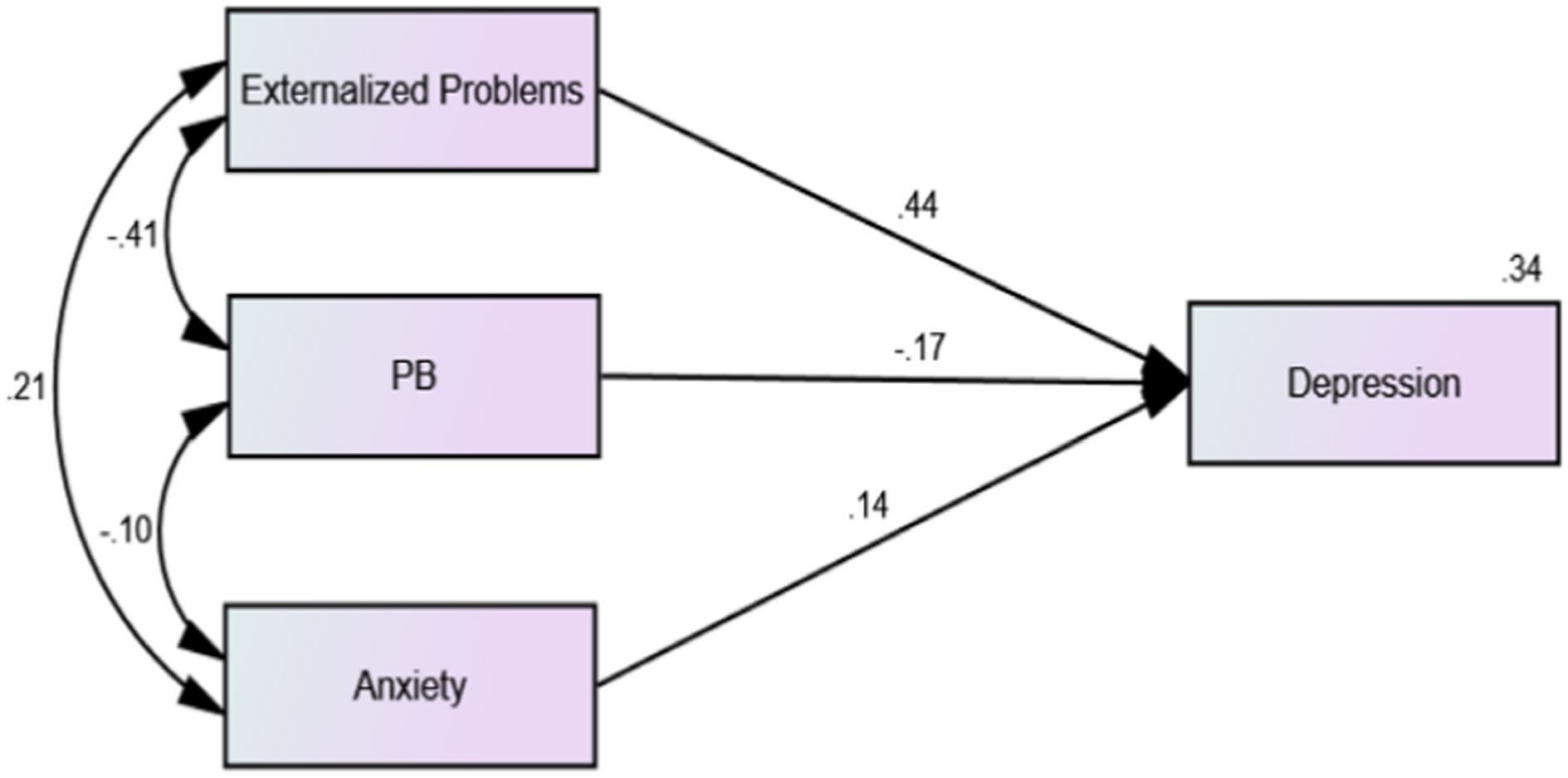
Regression model of external problems, prosocial behaviors, anxiety and depression factors of middle school students without anxiety symptoms.

**Figure 4:**
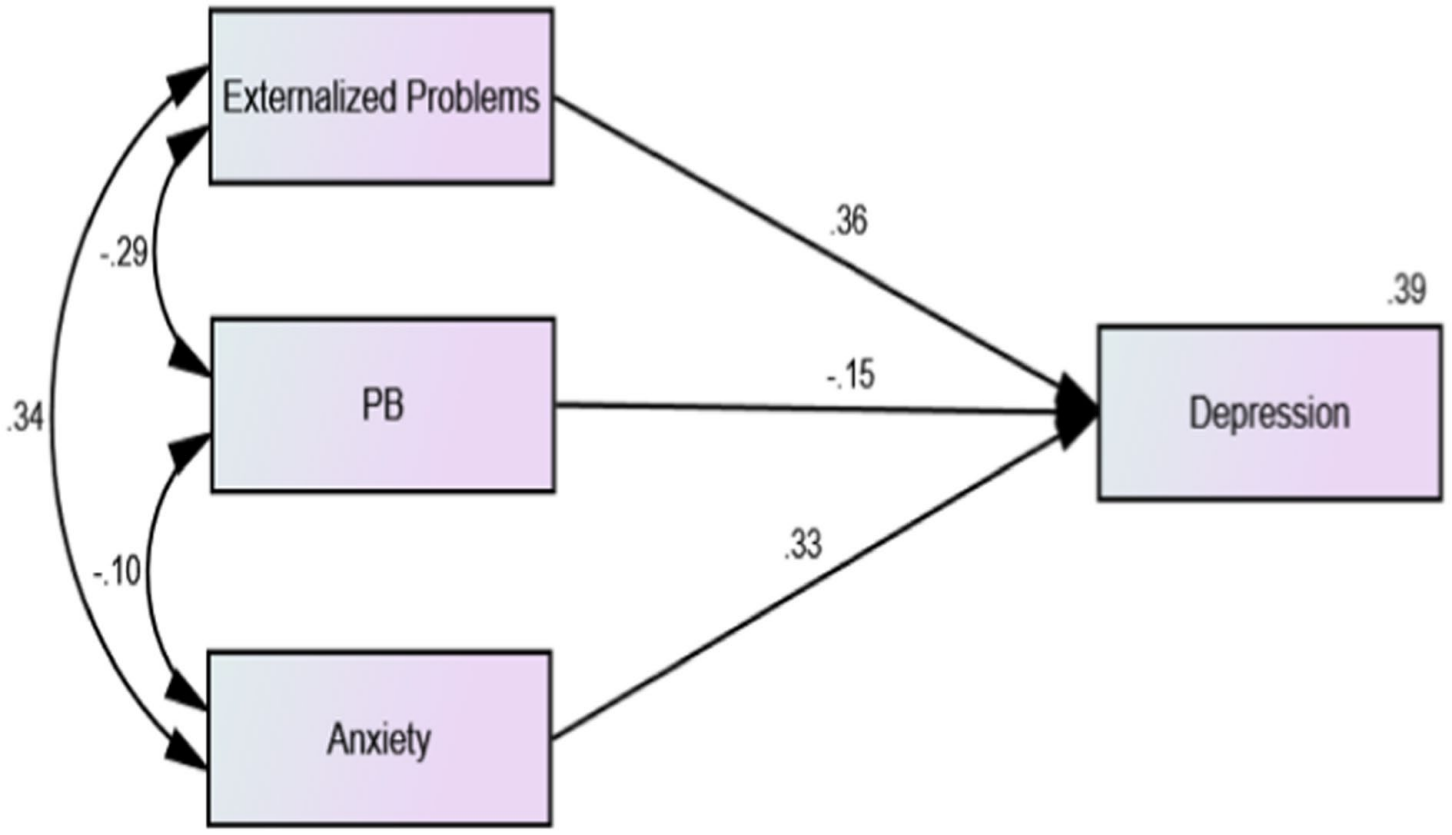
Regression model of external problems, prosocial behaviors, anxiety and depression factors of middle school students with anxiety symptoms.

The results showed that the overall model fit was acceptable (*χ*^2^/df = 7.754, RSMEA = 0.046, CFI = 0.990, IFI = 0.990, TLI = 0.964). The regression model showed that the correlations between adolescents’ externalizing problems and anxiety were significantly positive (*β* = −0.34, *p* < 0.001). As shown in Figure 1, among 2,639 middle school students without depressive symptoms, there was a significant negative correlation between prosocial behavior and anxiety (*β* = −0.11, *p* < 0.01), and prosocial behavior significantly negatively predicted the depressive symptom scores of middle school students (*β* = −0.17, *p* < 0.01). As shown in Figure 2, among the 530 middle school students with depressive symptoms, the correlation between prosocial behavior and anxiety was not statistically significant (*β* = −0.01, *p* > 0.01), and prosocial behavior did not significantly predict depressive symptoms in adolescents (*β* = −0.06, *p* > 0.01). As shown in Figures 1, 2, compared with adolescents without depression symptoms, adolescents with depressive symptoms did not exhibit a protective effect of prosocial behavior. While there was a significant negative correlation between prosocial behavior and anxiety symptoms among adolescents without depressive symptoms, this correlation was absent among adolescents with depressive symptoms. As shown in Figures 3, 4, there was no difference in the protective effect of prosocial behavior against depression between anxious and no anxious adolescents (*p* > 0.05).

## 4. Discussions

We found that anxiety and depression were prevalent among adolescents, with prevalence rates of 31.6 and 16.7%, respectively. Adolescence is a high-risk period for the onset of mood disorders, particularly anxiety and depression (40). Adolescence is a period of upheaval and change in physical, cognitive, emotional and social factors (41). During this period, adolescents face many developmental challenges, such as the need for academic achievement, peer relationships and independence from parents (42). Female adolescents are more likely than males to suffer from mood and anxiety disorders (43). Similar to previous studies, our findings confirmed that there was a gender difference in the distribution of anxiety, with female adolescents having a higher prevalence of anxiety than male adolescents. However, we did not find a gender difference in the incidence of depression. Previous research has suggested that female adolescents are biased toward depressive symptoms due to gender differences in hormone levels during puberty (44, 45). The gender difference in depression is one of the strongest findings in psychopathology research (46). Considerable empirical and theoretical research has been conducted on gender differences in depression. However, the findings have been inconsistent. One study found that male adolescents have a higher risk of depression than female adolescents (47). There may be a gender-age pattern of depressive symptoms. Several studies have shown that during childhood, boys are more likely than girls to suffer from depression (48). The increased prevalence of depression in females is thought to occur between the ages of 13 and 15 years (49, 50). The age range of our study sample was 11 to 16 years. Differences in the age range of samples may have contributed to the inconsistent results regarding gender differences in adolescent depression. In addition to age, differences in other nonphysical factors, such as research instruments, cultures, regions, and social structure, may also be important factors leading to inconsistent findings and a comprehensive meta-analysis of gender differences in adolescent depression could be conducted in the future. We also noted that living in extended families may protect adolescents’ emotional health compared to living in nuclear families. One possible explanation is that adolescents in nuclear families receive less emotional support than those in extended families, as these children can draw upon grandparents as additional (or alternative) sources of attachment, reassurance and knowledge and possibly experience decreased parental stress (51).

On the other hand, anxiety and depression are highly concurrent or sequential comorbidities. In some clinical samples, the correlation between dimensional measures of anxiety and depression symptoms was high (52), and the rate of diagnostic comorbidity was as high as 75% (11), with substantial comorbidity evident in both areas. We also found considerable levels of comorbid anxiety and depression in this large representative sample of Chinese adolescents. However, the comorbidity was commonly asymmetrical. That is, adolescents with a primary diagnosis of depression tended to have comorbid anxiety more often than adolescents with a primary diagnosis of anxiety experienced comorbid depression (13). This phenomenon was also confirmed in this study.

Most importantly, we analyzed the association between prosocial behavior and internalizing problems such as anxiety and depression. Our findings indicated that higher levels of prosocial behavior were associated with lower levels of anxiety and lower levels of depression. Specifically, prosocial behavior was negatively associated with anxiety and depression, consistent with some previous research (21–23), suggesting that prosocial behavior may prevent anxiety and depression. Prosocial behaviors are positive and friendly voluntary behaviors that an individual displays toward others. It is quite common during adolescence because during this period, opportunities for prosocial behaviors increase. Adolescents are especially sensitive to the quality and type of interactions they have with people in their social networks, and poor interpersonal interactions can lead to unfavorable mental health outcomes such as anxiety and depression (53). Previous research has shown that children who display high levels of prosocial behavior receive positive feedback and peer acceptance from their peers, and being liked and accepted by peers is associated with increased self-confidence and reduced anxiety and depression in children (54). Thus, prosocial behavior is important for forming and maintaining healthy relationships, and it can protect adolescents from maladjustment. Engaging in prosocial behaviors, especially with strangers, also promotes or stimulates the development of dominant personalities, which is especially important in early adolescence, as adolescents are developing character strengths that may continue to play a protective role in late adolescence and adulthood (55). In addition, according to response transformation theory, engaging in prosocial behaviors facilitates psychological adaptation through shifts in internal norms, values, and concepts of well-being as well as disengagement from self-focused psychological problems, such as anxiety and depression (56, 57). Prosocial behaviors, such as volunteering, leadership, and civic engagement, have often been incorporated into positive adolescent interventions, both as desired outcomes and as protective factors against future negative outcomes (58). All of the above findings suggest that adding prosocial behavior programs in schools may be beneficial. One school-based intervention study showed that when prosocial behavior was structurally added to the students’ curriculum, students’ mood level was significantly improved (59).

It is notable that the protective effect of prosocial behaviors against anxiety and depression may occur in both healthy and anxious adolescent populations. In further analyzes, we found that the protection of prosocial behavior against anxiety disappeared in individuals with depressive symptoms, but prosocial behavior still protected against depression in individuals with anxiety symptoms. Depression may have a greater impact on adolescents’ prosocial behaviors; they become more introverted, withdrawn and unable to exhibit warmth, compassion and helpfulness to others, resulting in decreased prosocial behavior. Individuals with low levels of prosocial behavior will experience more interpersonal problems and stress, making it difficult for them to stop negative self-thoughts and reflection and even aggravate these issues (60). This in turn increases the risk of depression and anxiety, resulting in a vicious cycle. Thus, treating depressive symptoms may be the first step for adolescents with depressive symptoms (e.g., through counseling interventions or medication). Alleviating the depressive symptoms of adolescents may help to restore the sensitivity provided by prosocial behavior and benefit from it.

Although our results indicated that prosocial behavior was negatively associated with depression and anxiety, these findings need to be verified in the future, especially in other adolescent samples. Inconsistent results have been reported in previous studies, particularly regarding the association between prosocial behavior and adolescent anxiety. For example, there is evidence that high levels of prosocial behavior protects against anxiety in girls (61). However, a recent meta-analysis showed that prosocial behavior was not related to anxiety (62). Another study showed that normative levels of anxiety were positively associated with prosocial behavior (63). In the future, research should focus on the association between prosocial behavior and adolescents’ internalizing problems from a more specific and multidimensional perspective.

## 5. Limitations

Our study has two potential limitations. First, it had a cross-sectional design; therefore, we could not dynamically analyze the association between prosocial behaviors and internalizing problems. Second, all measures in the study were self-reported, potentially leading to shared method bias. Although adolescents were considered the best reporters of their internal states (such as anxiety and depression), future research should investigate a wider range of reporters (e.g., parents, teachers).

## 6. Conclusion

Anxiety and depression are common in adolescents and are often comorbid. Adolescents spend more time in school than in any other formal institutional institution. Therefore, schools play a key role in the development of adolescents in all aspects. For adolescents without depressive symptoms, adding prosocial behavior to educational goals may be beneficial for their emotional management. Increasing school support and cultivating adolescents’ self-esteem and hope can promote prosocial behavior. However, in adolescents who already have depressive symptoms, increasing prosocial behavior may not be the most effective method. Instead, depressive symptoms may first need to be structurally addressed.

## Data availability statement

The raw data supporting the conclusions of this article will be made available by the authors, without undue reservation.

## Ethics statement

The studies involving humans were approved by the Ethics Committees of Shanghai Pudong New Area Mental Health Center. The studies were conducted in accordance with the local legislation and institutional requirements. Written informed consent for participation in this study was provided by the participants’ legal guardians/next of kin.

## Author contributions

XZ: Writing – original draft, Funding acquisition. TL: Data curation, Writing – original draft. GL: Methodology, Investigation, Software, Writing – review & editing. NZ: Investigation, Writing – original draft. XL: Formal analysis, Writing – original draft. YL: Software, Writing – original draft. YC: Writing – review & editing, Funding acquisition.

## References

[1] Zhao M Hu M. A multilevel model of the help-seeking behaviors among adolescents with mental health problems. Front Integr Neurosci. (2022) 16:946842. doi: 10.3389/fnint.2022.946842, PMID: 36118118 PMC9478167

[2] Cui Y Li F Leckman JF Guo L Ke X Liu J. The prevalence of behavioral and emotional problems among Chinese school children and adolescents aged 6-16: a national survey. Eur Child Adolesc Psychiatry. (2021) 30:233–41. doi: 10.1007/s00787-020-01507-6, PMID: 32172341

[3] Rutter M Kim-Cohen J Maughan B. Continuities and discontinuities in psychopathology between childhood and adult life. J Child Psychol Psychiatry. (2006) 47:276–95. doi: 10.1111/j.1469-7610.2006.01614.x, PMID: 16492260

[4] Patel V Flisher AJ Hetrick S McGorry P. Mental health of young people: a global public-health challenge. Lancet. (2007) 369:1302–13. doi: 10.1016/S0140-6736(07)60368-7 17434406

[5] Parodi KB Holt MK Green JG Porche MV Koenig B Xuan Z. Time trends and disparities in anxiety among adolescents, 2012–2018. Soc Psychiatry Psychiatr Epidemiol. (2021) 57:127–37. doi: 10.1007/s00127-021-02122-9, PMID: 34100110 PMC8183580

[6] Kessler RC Angermeyer M Anthony JC De Graaf R Demyttenaere K Gasquet I. Lifetime prevalence and age-of-onset distributions of mental disorders in the World Health Organization's world mental health survey initiative. World Psychiatry. (2007) 6:168–76. PMID: 18188442 PMC2174588

[7] Li J-Y Li J Liang J-H Qian S Jia R-X Wang Y-Q. Depressive symptoms among children and adolescents in China: a systematic review and Meta-analysis. Med Sci Monit. (2019) 25:7459–70. doi: 10.12659/MSM.916774, PMID: 31586039 PMC6792515

[8] Thapar A Collishaw S Pine DS Thapar AK. Depression in adolescence. Lancet. (2012) 379:1056–67. doi: 10.1016/S0140-6736(11)60871-4, PMID: 22305766 PMC3488279

[9] Essau CA. Comorbidity of depressive disorders among adolescents in community and clinical settings. Psychiatry Res. (2008) 158:35–42. doi: 10.1016/j.psychres.2007.09.007, PMID: 18164075

[10] Garber J Weersing VR. Comorbidity of anxiety and depression in youth: implications for treatment and prevention. Clin Psychol (New York). (2010) 17:293–306. doi: 10.1111/j.1468-2850.2010.01221.x, PMID: 21499544 PMC3074295

[11] Sørensen MJ Nissen JB Mors O Thomsen PH. Age and gender differences in depressive symptomatology and comorbidity: an incident sample of psychiatrically admitted children. J Affect Disord. (2005) 84:85–91. doi: 10.1016/j.jad.2004.09.003, PMID: 15620389

[12] Cummings CM Caporino NE Kendall PC. Comorbidity of anxiety and depression in children and adolescents: 20 years after. Psychol Bull. (2014) 140:816–45. doi: 10.1037/a0034733, PMID: 24219155 PMC4006306

[13] Axelson DA Birmaher B. Relation between anxiety and depressive disorders in childhood and adolescence. Depress Anxiety. (2001) 14:67–78. doi: 10.1002/da.1048, PMID: 11668659

[14] Ferdinand RF de Nijs PFA van Lier P Verhulst FC. Latent class analysis of anxiety and depressive symptoms in referred adolescents. J Affect Disord. (2005) 88:299–306. doi: 10.1016/j.jad.2005.08.004, PMID: 16182373

[15] Lewinsohn PM Rohde P Seeley JR. Adolescent psychopathology: III. The clinical consequences of comorbidity. J Am Acad Child Adolesc Psychiatry. (1995) 34:510–9. doi: 10.1097/00004583-199504000-00018, PMID: 7751265

[16] Karlsson L Pelkonen M Ruuttu T Kiviruusu O Heila H Holi M. Current comorbidity among consecutive adolescent psychiatric outpatients with DSM-IV mood disorders. Eur Child Adolesc Psychiatry. (2006) 15:220–31. doi: 10.1007/s00787-006-0526-7, PMID: 16502209

[17] Epkins CC Heckler DR. Integrating etiological models of social anxiety and depression in youth: evidence for a cumulative interpersonal risk model. Clin Child Fam Psychol Rev. (2011) 14:329–76. doi: 10.1007/s10567-011-0101-8, PMID: 22080334

[18] Walsh JJ Christoffel DJ Wu X Pomrenze MB Malenka RC. Dissecting neural mechanisms of prosocial behaviors. Curr Opin Neurobiol. (2021) 68:9–14. doi: 10.1016/j.conb.2020.11.006, PMID: 33278639 PMC8169714

[19] Penner LA Dovidio JF Piliavin JA Schroeder DA. Prosocial behavior: multilevel perspectives. Annu Rev Psychol. (2005) 56:365–92. doi: 10.1146/annurev.psych.56.091103.070141, PMID: 15709940

[20] Hay DF. Prosocial development. J Child Psychol Psychiatry. (1994) 35:29–71. doi: 10.1111/j.1469-7610.1994.tb01132.x, PMID: 8163628

[21] Adam SS Grant M. Doing good buffers against feeling bad: prosocial impact compensates for negative task and self-evaluations. Organ Behav Hum Decis Process. (2010) 111:13–22. doi: 10.1016/j.obhdp.2009.07.003

[22] Haroz EE Murray LK Bolton P Betancourt T Bass JK. Adolescent resilience in northern Uganda: the role of social support and prosocial behavior in reducing mental health problems. J Res Adolesc. (2013) 23:138–48. doi: 10.1111/j.1532-7795.2012.00802.x

[23] Fujiwara T. The role of altruistic behavior in generalized anxiety disorder and major depression among adults in the United States. J Affect Disord. (2007) 101:219–25. doi: 10.1016/j.jad.2006.11.024, PMID: 17222459

[24] Kovacs M. The Children's depression, inventory (CDI). Psychopharmacol Bull. (1985) 21:995–8. PMID: 4089116

[25] Watson HJ Egan SJ Limburg K Hoiles KJ. Normative data for female adolescents with eating disorders on the Children's depression inventory. Int J Eat Disord. (2014) 47:666–70. doi: 10.1002/eat.22294, PMID: 24797206

[26] Ivarsson T Svalander P Litlere O. The Children's depression inventory (CDI) as measure of depression in Swedish adolescents. A normative study. Nord J Psychiatry. (2006) 60:220–6. doi: 10.1080/08039480600636395, PMID: 16720513

[27] Wang L Feng Z Yang G Yang Y Dai Q Hu C. The epidemiological characteristics of depressive symptoms in the left-behind children and adolescents of Chongqing in China. J Affect Disord. (2015) 177:36–41. doi: 10.1016/j.jad.2015.01.002, PMID: 25745833

[28] Hale WW Raaijmakers Q Muris P Meeus W. Psychometric properties of the screen for child anxiety related emotional disorders (SCARED) in the general adolescent population. J Am Acad Child Adolesc Psychiatry. (2005) 44:283–90. doi: 10.1097/00004583-200503000-00013, PMID: 15725973

[29] Dirks MA Weersing VR Warnick E Gonzalez A Alton M Dauser C. Parent and youth report of youth anxiety: evidence for measurement invariance. J Child Psychol Psychiatry. (2014) 55:284–91. doi: 10.1111/jcpp.12159, PMID: 24552483

[30] Birmaher B Khetarpal S Brent D Cully M Balach L Kaufman J. The screen for child anxiety related emotional disorders (SCARED): scale construction and psychometric characteristics. J Am Acad Child Adolesc Psychiatry. (1997) 36:545–53. doi: 10.1097/00004583-199704000-00018, PMID: 9100430

[31] Rappaport BI Pagliaccio D Pine DS Klein DN Jarcho JM. Discriminant validity, diagnostic utility, and parent-child agreement on the screen for child anxiety related emotional disorders (SCARED) in treatment-and non-treatment-seeking youth. J Anxiety Disord. (2017) 51:22–31. doi: 10.1016/j.janxdis.2017.08.006, PMID: 28886420 PMC5761277

[32] Su L Wang K Fan F Su Y Gao X. Reliability and validity of the screen for child anxiety related emotional disorders (SCARED) in Chinese children. J Anxiety Disord. (2008) 22:612–21. doi: 10.1016/j.janxdis.2007.05.011, PMID: 17628391

[33] Goodman R Meltzer H Bailey V. The strengths and difficulties questionnaire: a pilot study on the validity of the self-report version. Eur Child Adolesc Psychiatry. (1998) 7:125–30. doi: 10.1007/s007870050057, PMID: 9826298

[34] Goodman R. The strengths and difficulties questionnaire: a research note. J Child Psychol Psychiatry. (1997) 38:581–6. doi: 10.1111/j.1469-7610.1997.tb01545.x 9255702

[35] Basel El-Khodary MS. The mediating role of trait emotional intelligence, prosocial behaviour, parental support and parental psychological control on the relationship between war trauma, and PTSD and depression. J Res Pers. (2019) 81:246–56. doi: 10.1016/j.jrp.2019.06.004

[36] Goodman R. Psychometric properties of the strengths and difficulties questionnaire. J Am Acad Child Adolesc Psychiatry. (2001) 40:1337–45. doi: 10.1097/00004583-200111000-00015 11699809

[37] Du Y Kou J Coghill D. The validity, reliability and normative scores of the parent, teacher and self report versions of the strengths and difficulties questionnaire in China. Child Adolesc Psychiatry Ment Health. (2008) 2:8. doi: 10.1186/1753-2000-2-8, PMID: 18445259 PMC2409296

[38] Michael RC Browne W. Alternative ways of assessing model fit. Sociol Methods Res. (1992) 21:230–58. doi: 10.1177/0049124192021002005

[39] Li-tze Hu PMB. Cutoff criteria for fit indexes in covariance structure analysis: conventional criteria versus new alternatives. Struct Equ Model Multidiscip J. (1999) 6:1–55. doi: 10.1080/10705519909540118

[40] Schacter HL Margolin G. When it feels good to give: depressive symptoms, daily prosocial behavior, and adolescent mood. Emotion. (2019) 19:923–7. doi: 10.1037/emo0000494, PMID: 30138009 PMC6387647

[41] Griffin A. Adolescent neurological development and implications for health and well-being. Healthcare. (2017) 5:62. doi: 10.3390/healthcare5040062, PMID: 28961184 PMC5746696

[42] Zarrett N Eccles J. The passage to adulthood: challenges of late adolescence. New Dir Youth Dev. (2006) 2006:13–28. doi: 10.1002/yd.179, PMID: 17225644

[43] Merikangas KR He J-p Burstein M Swanson SA Avenevoli S Cui L. Lifetime prevalence of mental disorders in U.S. adolescents: results from the National Comorbidity Survey Replication–Adolescent Supplement (NCS-A). J Am Acad Child Adolesc Psychiatry. (2010) 49:980–9. doi: 10.1016/j.jaac.2010.05.017, PMID: 20855043 PMC2946114

[44] Weller EB Kloos A Kang J Weller RA. Depression in children and adolescents: does gender make a difference? Curr Psychiatry Rep. (2006) 8:108–14. doi: 10.1007/s11920-006-0007-1, PMID: 16539885

[45] Paus T Keshavan M Giedd JN. Why do many psychiatric disorders emerge during adolescence? Nat Rev Neurosci. (2008) 9:947–57. doi: 10.1038/nrn2513, PMID: 19002191 PMC2762785

[46] Bebbington P. The origins of sex differences in depressive disorder: bridging the gap. Int Rev Psychiatry. (2009) 8:295–332. doi: 10.3109/09540269609051547

[47] Verboom CE Sijtsema JJ Verhulst FC Penninx BW Ormel J. Longitudinal associations between depressive problems, academic performance, and social functioning in adolescent boys and girls. Dev Psychol. (2014) 50:247–57. doi: 10.1037/a0032547, PMID: 23566082

[48] Cyranowski JM Frank E Young E Shear MK. Shear MK adolescent onset of the gender difference in lifetime rates of major depression: a theoretical model. Arch Gen Psychiatry. (2000) 57:21–7. doi: 10.1001/archpsyc.57.1.21, PMID: 10632229

[49] Hankin BL Abramson LY Moffitt TE Silva PA McGee R Angell KE. Development of depression from preadolescence to young adulthood: emerging gender differences in a 10-year longitudinal study. J Abnorm Psychol. (1998) 107:128–40. doi: 10.1037/0021-843X.107.1.128, PMID: 9505045

[50] Twenge JM Nolen-Hoeksema S. Age, gender, race, socioeconomic status, and birth cohort difference on the children's depression inventory: a meta-analysis. J Abnorm Psychol. (2002) 111:578–88. doi: 10.1037/0021-843X.111.4.578, PMID: 12428771

[51] Hwang HJ St James-Roberts I. Emotional and behavioural problems in primary school children from nuclear and extended families in Korea. J Child Psychol Psychiatry. (1998) 39:973–9. doi: 10.1111/1469-7610.00400, PMID: 9804030

[52] Stark KD Laurent J. Joint factor analysis of the Children's depression inventory and the revised Children's manifest anxiety scale. J Clin Child Psychol. (2001) 30:552–67. doi: 10.1207/S15374424JCCP3004_11, PMID: 11708242

[53] Masten CL Eisenberger NI Borofsky LA Pfeifer JH McNealy K Mazziotta JC. Neural correlates of social exclusion during adolescence: understanding the distress of peer rejection. Soc Cogn Affect Neurosci. (2009) 4:143–57. doi: 10.1093/scan/nsp007, PMID: 19470528 PMC2686232

[54] TS BRB Rubin-Smith J Fitzmaurice GM Gilman SE. Sierra Leone's former child soldiers: a longitudinal study of risk, protective factors, and mental health. J Am Acad Child Adolesc Psychiatry. (2010) 49:606–15. doi: 10.1016/j.jaac.2010.03.008, PMID: 20494270 PMC3157024

[55] Padilla-Walker LM Millett MA Memmott-Elison MK. Can helping others strengthen teens? Character strengths as mediators between prosocial behavior and adolescents’ internalizing symptoms. J Adolesc. (2020) 79:70–80. doi: 10.1016/j.adolescence.2020.01.001, PMID: 31926448

[56] CE SMS. Helping others helps oneself: response shift effects in peer support. Soc Sci Med. (1999) 48:1563–75. doi: 10.1016/S0277-9536(99)00049-0, PMID: 10400257

[57] MA SCS. Integrating response shift into health-related quality of life research: a theoretical model. Soc Sci Med. (1999) 48:1507–15. doi: 10.1016/S0277-9536(99)00045-3, PMID: 10400253

[58] Ciocanel O Power K Eriksen A Gillings K. Effectiveness of positive youth development interventions: a meta-analysis of randomized controlled trials. J Youth Adolesc. (2016) 46:483–504. doi: 10.1007/s10964-016-0555-6, PMID: 27518860

[59] Berger R Benatov J Cuadros R VanNattan J Gelkopf M. Enhancing resiliency and promoting prosocial behavior among Tanzanian primary-school students: a school-based intervention. Transcult Psychiatry. (2018) 55:821–45. doi: 10.1177/1363461518793749, PMID: 30091688

[60] Fehr E Fischbacher U. The nature of human altruism. Nat Rev Neurosci. (2003) 425:785–91. doi: 10.1038/nature02043, PMID: 14574401

[61] Flynn E Ehrenreich SE Beron KJ Underwood MK. Prosocial behavior: long-term trajectories and psychosocial outcomes. Soc Dev. (2015) 24:462–82. doi: 10.1111/sode.12100, PMID: 26236108 PMC4517683

[62] Memmott-Elison MK Holmgren HG Padilla-Walker LM Hawkins AJ. Associations between prosocial behavior, externalizing behaviors, and internalizing symptoms during adolescence: a meta-analysis. J Adolesc. (2020) 80:98–114. doi: 10.1016/j.adolescence.2020.01.012, PMID: 32087386

[63] Padilla-Walker LM Carlo G Nielson MG. Does helping keep teens protected? Longitudinal bidirectional relations between prosocial behavior and problem behavior. Child Dev. (2015) 86:1759–72. doi: 10.1111/cdev.12411, PMID: 26511897

